# Efficient Execution of Complex Context Queries to Enable Near Real-Time Smart IoT Applications

**DOI:** 10.3390/s19245457

**Published:** 2019-12-11

**Authors:** Alireza Hassani, Alexey Medvedev, Arkady Zaslavsky, Pari Delir Haghighi, Prem Prakash Jayaraman, Sea Ling

**Affiliations:** 1 School of Information Technology, Deakin University, Geelong 3216, Australia; alexey.medvedev@deakin.edu.au (A.M.); arkady.zaslavsky@deakin.edu.au (A.Z.); 2 Faculty of Information Technology, Monash University, Melbourne 3145, Australia; pari.delirhaghighi@monash.edu (P.D.H.); chris.ling@monash.edu (S.L.); 3 Department of Computer Science and Software Engineering, Swinburne University of Technology, Melbourne 3122, Australia; pjayaraman@swin.edu.au

**Keywords:** complex, context, query, execution, IoT, CMP

## Abstract

As the Internet of Things (IoT) is evolving at a fast pace, the need for contextual intelligence has become more crucial for delivering IoT intelligence, efficiency, effectiveness, performance, and sustainability. Contextual intelligence enables interactions between IoT devices such as sensors/actuators, smartphones and connected vehicles, to name but a few. Context management platforms (CMP) are emerging as a promising solution to deliver contextual intelligence for IoT. However, the development of a generic solution that allows IoT devices and services to publish, consume, monitor, and share context is still in its infancy. In this paper, we propose, validate and explain the details of a novel mechanism called Context Query Engine (CQE), which is an integral part of a pioneering CMP called Context-as-a-Service (CoaaS). CQE is responsible for efficient execution of context queries in near real-time. We present the architecture of CQE and illuminate its workflows. We also conduct extensive experimental performance and scalability evaluation of the proposed CQE. Results of experimental evaluation convincingly demonstrate that CoaaS outperforms its competitors in executing complex context queries. Moreover, the advanced functionality of the embedded query language makes CoaaS a decent candidate for real-life deployments.

## 1. Introduction

Nowadays, advancements in hardware and software technologies have made it possible to embed sensing, computation, and communication capabilities in everyday objects, from a coffee mug to an autonomous car, and turn them into smart connected objects. These devices can sense enormous amounts of data about their environment and share it via the Internet. This network of inter-connected devices, known as the Internet of Things (IoT), is a fast-evolving trend. It is expected the overall spending on IoT will reach US $1.3 trillion by 2020 from US $696 billion in 2015 [1] and the number of devices connected to the internet will reach 20 to 30 billion in 2020.

The proliferation of such IoT devices offers the enormous potential to share rich, useful and relevant information about the environment that can help in the development of smart services. These services, which we refer to as IoT services, enable that development of many smart applications in domains, such as smart cities, smart environment, smart agriculture, and eHealth. A key requirement to deliver the smartness required by the application lie in the ability to extract context from the data produced by IoT devices. Context, as defined by Dey, is “any information that can be used to characterise the situation of an entity, where an entity is a person, place, or object that is considered relevant to the interaction between a user and an application, including the user and applications themselves” [2]. Such IoT applications that utilise context data and adapt their behaviours accordingly are known as context-aware IoT applications [3]. Context-awareness enables intelligent adaptation of IoT applications such that they can perform their tasks in an efficient, proactive and autonomous manner.

For example, consider a smart home scenario where a smart washing machine is tasked to wash a piece of clothing tagged with information (e.g., using RFID) regarding fabric care instructions. Using this information, the smart washing machine can automatically choose the right setting for washing the clothes. Moreover, this information can be used by a smart tumble dryer to decide what temperature and revolutions per minute (RPM) should be used for drying the clothes. Assuming the delicate clothing material is not suitable for tumble drying, without context, the smart dryer will dry the clothes unaware of this fact. Augmenting IoT application with context that stem from IoT devices will enable the application (e.g., an application running on the smart dryer) to reason about the data and arrive at the right decision, in this case, not to tumble dry the delicate clothes.

Context can have different levels of abstraction such as low-level information—a temperature value of 35 °C, or high-level context, which is inferred from low-level context such as ‘a fire threat’. High-level context is also known as ‘situation’. While context-driven intelligence is a fundamental factor for IoT sustainability, growth, interoperability and acceptance, IoT’s characteristics, such as the unprecedented scale, volume of data, heterogeneity and dynamism, will make the development of context-aware IoT applications and services a very challenging task.

In general, three typical approaches exist for the development of context-aware applications [4]. In the first approach, context-aware applications acquire, process and use their context of interest themselves. In the second approach, context-aware applications are developed by using some libraries or toolkits that facilitate obtaining and processing context. In the third approach, the context-aware applications are developed on the basis of context-aware middleware that enables context management (i.e., acquire, process, store, and publish). The third approach is communication through a centralised middleware that offers all the required functionality for context management. This approach has several advantages compared to the first and second approaches. First of all, it can reduce the complexity of developing context-aware IoT applications as all the context-related functions are handled by the middleware [4]. Secondly, using a central middleware for developing context-aware IoT applications enables context exchange across IoT silos [5]. Further, the greater benefit is in being able to share the context extracted/reasoned from data produced by the IoT devices with other IoT applications that can use this context to support decision making, actuation, analysis etc [6]. Therefore, we believe using such type of middleware, which is referred to as Context Management Platform (CMP), for the development of context-aware IoT applications are superior to the first two approaches. 

A fundamental requirement of a CMP is to be able to provide support for publishing, querying, monitoring, and sharing contextual information. Such a platform will manage the interaction between the sources of context and offer contextual information to context-aware IoT applications. A notable number of CMPs have been proposed [7,8,9,10]. However, the existing CMPs suffer from one common shortcoming, which is the lack of a generic and expressive interface that allows IoT devices, applications, and services to publish, consume, monitor, and share context data seamlessly. To address this shortcoming, in our earlier research, we have proposed a high-level language for querying context [6,11,12], which called Context Definition and Query Language (CDQL). 

CDQL allows IoT devices and applications to query, monitor, and consume the context data produced by IoT devices and services. One of the main advantages of CDQL compared to existing context query languages, is its ability to express complex context queries. Existing CQLs have very limited support for querying high-level context and providing situation-awareness. More importantly, most of the existing CQLs can only support a single entity per query. More precisely, it is not possible in existing CQLs to query about context of multiple joined entities in a single query. In contrast, CDQL supports querying high-level context as well as querying multiple entities in a single query, which makes CDQL queries complex. Offering such functions will reduce the development complexity of context-aware applications. Moreover, supporting complex context queries can lead to higher performance by reducing communication overhead and creating the opportunity for query optimisation [13]. However, in order to support complex queries, it is required to develop a more complicated query execution mechanism for CMP to handle such queries.

In continuation of our efforts towards operationalising and externalising context for smart IoT applications, in this paper, we propose, develop, implement, and evaluate a comprehensive and efficient mechanism for enabling execution of context queries in IoT ecosystem in near real-time. We call this mechanism Context Query Engine (CQE). CQE is capable of processing and executing context queries in a resource and time-efficient manner. This work is part of EU Horizon-2020 project called bIoTope (www.biotope-h2020.eu)—Building IoT OPen Innovation Ecosystem for connected smart objects where Context-as-a-Service (CoaaS) [14] forms an important part of its service offerings.

The main contributions of this paper are summarised below:

- We have designed and developed a mechanism (i.e., Context Query Engine) that allows execution of complex context queries in dynamic IoT ecosystems.
- We have implemented a prototype of the proposed solution.
- We have conducted a comprehensive set of experiments to measure the performance of CQE in the execution of various context queries and compare its performance with a well-known existing CMP (i.e., Fiware Orion).

This paper is organised as follows: Section 2 summarises the main research directions and the related work in the area. Section 3 describes the high-level architecture of the CoaaS platform as well as the foundations of CDQL. It also sets the main terminology and definitions. Section 4 presents the architecture and underlying components and algorithms of CQE. Section 5 describes the implementation details of the proposed solution. Section 6 is devoted to performance evaluation of CQE. Section 7 concludes the paper and sets directions for future work.

## 2. Related Works

The management and provisioning of context information are essential elements for realising context-aware services and applications in the realm of IoT. In this section, we first review the main aspects and functionalities of a CMP. Then, a brief overview of some of the most recognised CMPs is presented.

The major functionalities of context management platforms can be subdivided into six classes [10], which are below:

- **Sensor Data Acquisition.** This function is responsible for fetching raw context data from multiple sources. In the context-aware system, it is essential that the system can support a variety of heterogeneous context sources. Based on the computational capability of context sources, pre-processing and data cleaning might be executed locally (on the context source) or externally as a part of the CMP’s functionality.
- **Context Storage.** This function refers to the mechanism of persisting contextual information in the platform. Two crucial aspects of context storage are: (i) the storage of current context (context caching) and (ii) the storage of historical context. Caching improves the performance of CMPs in answering incoming queries by omitting the process of fetching repeated context. Moreover, a CMP should be capable of storing and indexing historical context. Historical context can be utilised by CMPs to produce valuable insights about IoT entities. For example, the historical data can be used to learn the habits of IoT entities and predict their future states. This can be used both for query serving and platform’s self-adaptation.
- **Context Service registration and Discovery.** A CMP should provide a mechanism that allows sources of context (i.e., IoT devices and services) to describe and register their offered contextual information. Moreover, it is vital for a CMP to be able to search and find the matching sources of context for an incoming query.
- **Privacy, Security & Access Control.** This feature is considered as a vital function in CMPs as they might expose sensitive information about IoT devices and their owners to unauthorised third parties. As a result, it is essential for a CMP to have a sophisticated authentication and authorisation mechanism to guarantee the privacy and security of users’ contextual information.
- **Context Processing & Reasoning.** Sources of context (e.g., sensors) mostly offer raw sensory data to CMPs. Hence, a CMP is required to perform some pre-processing to infer context information from raw sensory data. Moreover, in many use-cases, it is essential to infer high-level context/situation from multiple existing low-level context. Therefore, a CMP should be capable of performing different context inference and situation reasoning techniques such as feature extraction, rule-based reasoning or probabilistic inference.
- **Context Querying (Context Diffusion & Distribution).** The ultimate objective of a CMP is to facilitate the development of context-aware applications. Each context-aware application has unique contextual requirements. As a result, a CMP should provide a generic approach that allows context-aware applications to request for contextual data based on their unique requirements. This approach should define a comprehensive and flexible query language that allows context-aware applications to query the context of their entities of interest. Moreover, it should support different communication modes, namely push-based queries and pull-based queries. Push-based queries refer to event-driven asynchronous queries (i.e., publish/subscribe) that allows context-aware applications to subscribe for changes in the context of their entities of interest and get notified about context changes. Pull-based queries refer to synchronous on-demand queries.

Existing context management platforms can be classified into three main generations. The earliest generation, such as the Active Badge System [15], only focused on utilising location data. The second generation includes systems such as Context Toolkit [2], SOCAM [16], and CoBrA [17]. These platforms tried to achieve a higher level of generality, supporting more varieties of context. However, these platforms suffer from several common constraints that make them inefficient to be used in real-world context-aware systems. For example, Context Toolkit does not support context sharing among heterogeneous context providers and consumers as it is not built on a foundation of common ontologies with explicit semantic representation [18]. Another example of these constraints can be seen in the SOCAM platform. SOCAM adopted First Order Logic (FOL) for supporting context/situation reasoning. While FOL is useful to infer basic context, it does not support reasoning under uncertainty. To conclude, the main constraints of the second-generation platforms are the lack of fault tolerance and scalability, poor interoperability support and naïve context/situation reasoning and processing.

The effort of the research community to address these limitations lead to the development of third-generation context management platforms, such as Context-Aware Services Framework (CA4IoT) [19], CAMPUS [20], and Fiware Orion Context Broker [21]. 

CA4IOT is a multi-layered IoT platform that provides a mechanism that helps users to select the most suitable sensors for a specific task. CA4IOT has four primary layers, which are SDAL, CSDL, CPRL, and DSCDL. SDAL is in charge of context acquisition. CSDL is responsible for automatic discovery of context services. CPRL provides a set of methods that allows the processing of context data. Lastly, DSCDL is in charge of user management. While the authors CA4IoT did a great job in explaining the high-level architecture of their platform, they did not provide any details about technical challenges and implementation details of this middleware. Therefore, the validity and feasibility of their proposed platform are not assessable. 

Context-Aware Middleware for Pervasive and Ubiquitous Service (CAMPUS) [20] is another CMP that proposed to facilitate the development of context-aware applications. CAMPUS is designed on the basis of three main techniques, which are a compositional adaptation, ontology, and description logic/first-order logic reasoning. The main feature of CAMPUS platform is its decision-making mechanism that enables automatic adjustment to the real-time changes of context in context-aware applications.

Among the existing CMPs, we found Orion Context Broker [21] the most advanced platform in terms of consistent development and market penetration. Orion is one of the core components of Fiware platform. Fiware Orion allows management of the entire lifecycle of context information including updates, queries, registrations and subscriptions. Orion Context Broker is developed on top of a CQL called NGSI v2 [22]. NGSI v2 defines a REST API based on the standard usage of HTTP verbs and enables context consumers to access context data simply through making HTTP requests. NGSI was recently used as a base for the development of an ETSI NGSI-LD standard for context information management [23,24]. However, the NGSI language suffers from a number of drawbacks. NGSI supports only one entity per query, which limits the expressivity, flexibility, and query performance, and it also adds network overhead. Moreover, NGSI has limited support for situation reasoning and monitoring. To address this, FIWARE has integrated the Esper Complex Event Processing (CEP) engine [25], which uses Esper EPL [26] to represent monitored situations. However, NGSI and Esper EPL are two disjoint technologies, and this increases the development and maintenance efforts. Such an approach also adds conceptual complexities as Esper EPL is a more generic technology and is not designed to support IoT context-aware environments.

As discussed above, the third generation CMPs successfully addressed some of the main limitations of the earlier generation of context management platforms. However, we believe the development of a comprehensive CMP that can evolve to an industry-standard level is still in its infancy. We believe one of the main shortcomings of these middleware systems is the lack of a comprehensive and flexible context query language (CQL) that allows context-aware applications to search and retrieve contextual data based on their specific requirements [12]. The CQLs supported by existing CMPs are not expressive enough to represent complex context queries. For example, to the best of our knowledge, none of the existing CQLs supports querying multiple entities in one query. Eventually, none of these languages has become a widely adopted standard, while such a standard is very important nowadays [23]. 

Another important shortcoming of existing CMPs is their limited support for context processing, including context reasoning and situation inferences. We believe to deliver the promised smartness, just storing and sharing the context data is not sufficient. It is essential to process the context data, which is retrieved from multiple sources, to produce high-level context, infer the real-time situation of an entity of interest and its surroundings, and react to it accordingly. 

The last but not least, the performance and scalability of the existing CMPs are still far from the industry standards level, where CMPs should store, process, and monitor context of millions of IoT entities in near real-time. In the rest of this paper, as a step towards maturing the context management platforms, we will propose a novel mechanism that enables efficient execution of complex context queries in near real-time.

## 3. Context-as-a-Service Background

This section describes the overview of CoaaS platform and its role in the IoT ecosystem. Furthermore, the formal definitions for the underlying concepts of CoaaS will be presented in this section. Lastly, we present the blueprint architecture of CoaaS platform and briefly explain its main components.

### 3.1. Definitions

In this section, we introduce the fundamentals and definitions of the Context-as-a-Service (CoaaS) platform. CoaaS is a context management platform, which has been designed to facilitate the development of context-aware IoT applications by providing a generic yet flexible mechanism to query and publish context. In other words, CoaaS enables applications to provide and consume context about their entities of interest seamlessly, without requiring manual integration of IoT silos.

As mentioned earlier, context is the information that can be used to characterise the situation of an entity [2]. Entities can be persons, locations, or objects which are considered to be relevant for the behaviour of an application. An entity can be characterised by a set of parameters, known as context attributes:

**Definition 1 (Entity and Context Attribute).**  *In context-aware systems, an entity (denoted by E) accounts for a physical or virtual object (such as a person, a car, an electronic device, or an event) that can be associated with one or more context attributes (denoted by ca, which can be any type of data that characterises this entity.*

For example, a ‘car’ entity can have a location, speed, fuel level, the number of available seats, model, and manufacturer as its context attributes. 

**Figure 1:**
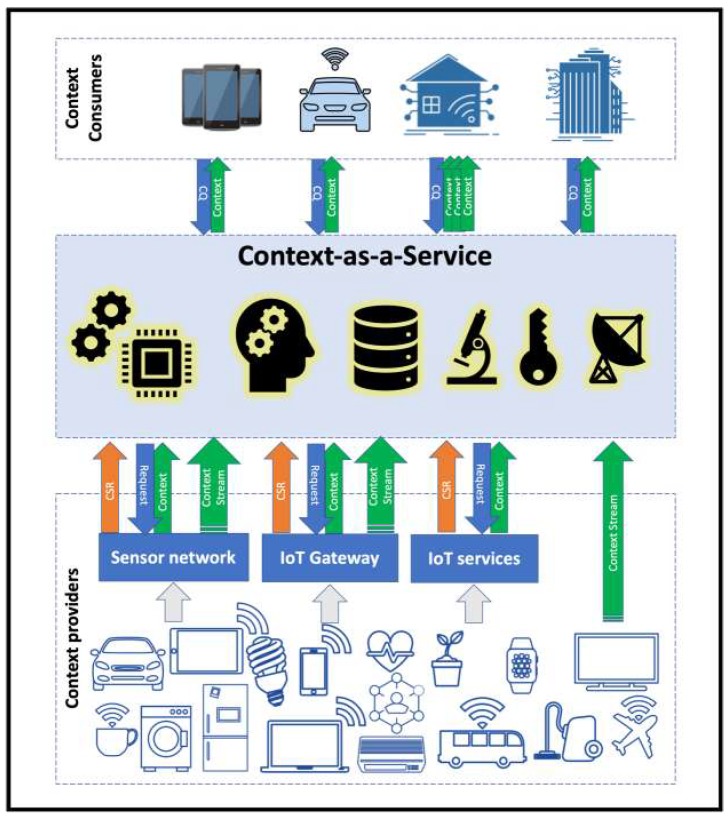
Overview of the Context-as-a-Service platform in the IoT ecosystem.

The big-picture view of Context-as-a-Service platform in the IoT ecosystem is represented in Figure 1, which consists of three layers of Context Consumers, Context Providers, and the Context Management Platform (CMP). 

The top layer is a collection of context-aware IoT applications in various domains that require contextual information in order to perform their task. These applications are interested in collecting contextual information about a particular entity with specific characteristics. They are defined as context consumers.

**Definition 2 (Context Consumer).**  *Context Consumer (CC) refers to any device or system that queries and receives context about one or several entities.*

The bottom layer, in Figure 1 shows the sources of context, which consists of sensors, smart connected devices, and systems that can produce context about entities. They are the context providers.

**Definition 3 (Context Provider).**  *Context Provider (CP) refers to any device, application or system that provides context or data that can be used to infer context about one or several entities.*

We distinguish between different classes of CPs based on the type of context they produce. At the most basic level, a context provider can be a standalone sensor that is connected to the Internet and is capable of transmitting raw sensory data about a particular attribute of an entity. For example, a temperature sensor connected to a Wi-Fi microchip such as ESP8266 [27] can act as a CP. However, CPs can be more sophisticated and provide either low-level or high-level context about characteristics of several IoT entities. For example, IoT gateways and middleware, sensor networks, or even a mobile application can play the role of a CP and supply context. Lastly, some web-based services such as Google Maps APIs, or weather forecast APIs can also act as context providers as they can produce useful information. As a result, based on the CPs’ class, each context provider can have one or more services, which produce context about an entity. We refer to these services as Context Services.

**Definition 4 (Context Service).**  *A Context Service (denoted by $cs_{j,\;j\;\in \;\mathbb{N}}$) provides contextual information about a particular entity. Context service can be represented as a triple: 〈E, CA, P〉 where E denotes the related entity, CA is a set of provided context attributes, and Predicates (denoted by P form a composite logical expression defined over CA.*

For example, a smart garage (which is a context provider) can provide a context service to deliver values of context attributes such as cost, available facilities, and time limit (contextual information) about available car parks (entity) in a specific location. Further, the working hours of this garage are from 8 am to 8 pm during weekdays, and 10 am to 10 pm on weekends (complex context attribute). This context service description can be represented as:

$$cs_{1}:\;\langle E_{1},\;CA_{1},\;P_{1}\rangle$$

where:

$$\left\{ \begin{array}{c} E_{1}:carpark\\ CA_{1}:\left\{\cos t,\;\mathrm{location},\;\mathrm{available}\;\mathrm{facilities},\;\mathrm{number}\;\mathrm{of}\;\mathrm{available}\;\mathrm{parking}\;\mathrm{spots},\mathrm{working}\;\mathrm{hours}\right\}\\ P_{1}:\\ location=LocA\;\wedge \\ (\left(workingHours\;between\;8:00\;and\;20:00\;\wedge weekdays\right)\vee \\ \left(workingHours\;between\;10:00\;and\;22:00\;\wedge weekends\right)) \end{array}\right.$$

On the basis of the presented definition for context services, we have designed a high-level language called CSDL for describing context services, which is presented in [6].

In Figure 1, the middle layer shows the CoaaS platform, which enables global standardisation and interworking among context providers and consumers. CoaaS can interact with CPs in two ways, either by fetching the contextual data on-demand or through processing the incoming data streams. In the first case, the CPs must have registered the description of their services first by sending a context service registration (CSR) request. Then, CoaaS can retrieve data about IoT entities by sending requests to corresponding providers on-demand. As mentioned above, CoaaS can also process streams of context updates, which CPs are sending to the platform. Context updates contain updates of the entities’ states and are processed by CoaaS to monitor situations. The blueprint architecture of CoaaS platform is presented in Section 3.2.

On the other hand, context consumers can retrieve context information from the middleware by issuing context queries (CQ).

**Definition 5 (Context Query).**  *Context query is a request for contextual information (either context attributes or high-level context inferred from context attributes) from one or many entities.*

For example, a smart vehicle can issue a context query to retrieve the cost, location, and the number of available spaces (contextual information) of the best parking facilities (the entity of interest) near the driver’s meeting location based on his/her preferences. This query contains three main entities, namely parking facility, smart vehicle, and driver. 

Each context query can be split into several sub-requests, where the final result of the query will be computed based on the contextual information retrieved from the results of these sub-requests by aggregating the results or using the results to infer a higher-level context.

**Definition 6 (Context Request).**  *A context request (denoted by $cr_{i,\;i\in \;\mathbb{N}}$) represents a request for contextual information about a particular entity. Context request can be represented as a triple: 〈E, CA, P〉 where E denotes the entity of interest, CA is a set of requested context attributes, and P. is a set of predicates, which are defined over CA using logical expressions.*

Based on Definitions 5 and 6, we have designed a novel context query language that supports complex context queries concerning various entities. This language is exhaustively presented in [11].

The aforementioned context query for finding car parks can be broken down into three context requests, one for each entity. The first request is issued to retrieve context about the driver, the second request is issued to identify the smart vehicle, and the last context request is issued to retrieve information about available parking. The formal descriptions of context requests are represented as below, and the actual CDQL representation of the query is shown in Code block 1:

$$cr_{1}:\;\langle person,\;\left\{meeting,\;parking\;preferences\right\},\;\left\{driver\;id=101\right\}\rangle$$

$$cr_{2}:\;\langle car,\;\left\{location,\;width,\;height,\;length\right\},\;\left\{VIN=202\right\}\rangle$$

$$cr_{3}:\;\langle \begin{array}{c} parking\;facility,\;\left\{location,\;cost,\#available\;spots\right\},\\ \left\{distance\;\left(meeting.location,parking.location\right)<500\right\} \end{array}\rangle$$

After defining the underlying concepts in this section, we present in the next section the blueprint architecture of CoaaS platform and introduce its main components and data flows.

### 3.2. CoaaS Platform Blueprint Architecture

**Figure 2:**
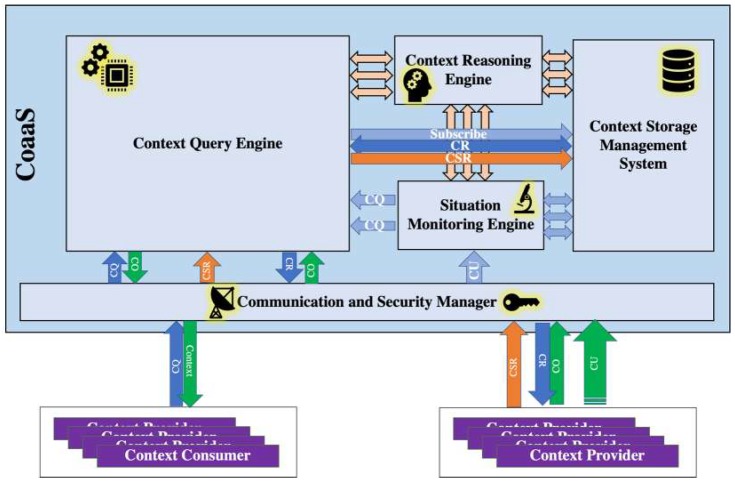
CoaaS Blueprint Architecture.

This section presents the blueprint architecture of the CoaaS platform and discusses its main components. As mentioned in Section 2, CMPs have six major functionalities, namely (i) sensor data acquisition, (ii) context storage, (iii) context lookup and discovery, (iv) privacy, security and access control, (v) context processing and reasoning, and (vi) context diffusion and distribution. Aligned with these functionalities, we designed the blueprint architecture of CoaaS platform accordingly, which can be seen in Figure 2. 

As this figure shows, the CoaaS platform has five main components: Communication and Security Manager, Context Query Engine (CQE), Situation Monitoring Engine (SME), Context Storage Management System (CSMS), and Context Reasoning Engine (CRE).

Table 1 provides a mapping between the CoaaS components and the aforementioned CMP functionalities. In the rest of this section, a brief description of each of these main enabling components is presented.

The Communication Manager is responsible for the initial handling of all incoming and outgoing messages, namely context services registration (CSR), context queries (CQ), context updates (CU), and context responses. This module acts as a proxy and distributes all the incoming messages from CPs and CCs to the corresponding components. To guarantee the privacy and security of CoaaS, this component is linked to the Security Manager. The Security Manager module firstly checks the validity of incoming messages and authenticates requests. Moreover, the Security Manager checks whether the context consumer has access to the requested context service or not (authorization). Lastly, it is also responsible for monitoring all the incoming messages to identify any suspicious patterns, such as distributed denial-of-service (DDoS) attacks.

The Context Query Engine (CQE) is mainly responsible for parsing the incoming queries, generating and orchestrating the query execution plan, and producing the final query result. Furthermore, this component also takes care of fetching required data from context providers on demand. This component will be discussed in more detail in Section 4.

The Situation Monitoring Engine (SME) is designed to support the continuous monitoring of incoming context, infer situations from available context, detect changes in situations and provide notification of detected changes. This component monitors the real-time context of the IoT entities and reason about their situations. It also initiates the actuation procedure by notifying context consumers when their situation of interest is detected. 

The Context Storage Management System (CSMS), which is described in detail in [28], has three main objectives. First of all, it stores descriptions of context services and facilitates service discovery. Secondly, it caches contextual information to ensure reasonable query response time and deals with problems like network latencies and potential unavailability of context sources. The third objective is storing and analysing the historical context to facilitate self-adaptation and efficiency optimization.

The main task of the Context Reasoning Engine (CRE) is to infer situations from raw sensory data or existing primitive low-level context. It is a common need in many context-aware IoT applications to query about the situation of a context entity or trigger a query when a specific situation is detected. A situation can be seen as a high-level context that is inferred from multiple low-level context [29]. 

In the next subsection, we will briefly describe the structure and syntax of CDQL query language. 

### 3.3. Context Definition and Query Language (CDQL)

**Figure 3:**
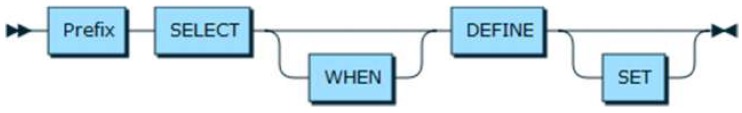
CQL Production Rule.

Context Definition and Query Language (CDQL) [7,8,9] is a flexible and generic context query language that allows IoT applications to publish and query context. Figure 3 illustrates the production rule of the CDQL language, which consists of three mandatory and two optional clauses.

The mandatory clauses are PREFIX, SELECT, and DEFINE. The PREFIX clause is responsible for identifying the semantic vocabularies that are used in the query to facilitate interoperability. The SELECT clause is responsible for identifying the output of the query which can be either low-level context (a set of context attributes), or high-level context. The DEFINE clause is considered as the core of CDQL and makes it possible to write complex queries that include various entities and constraints. This clause defines the entities that are involved in a context query. Code block 1 provides an example of a basic CDQL query. This query expresses a request to find available car parks and can be issued by a smart-car or the navigation system’s backend server.

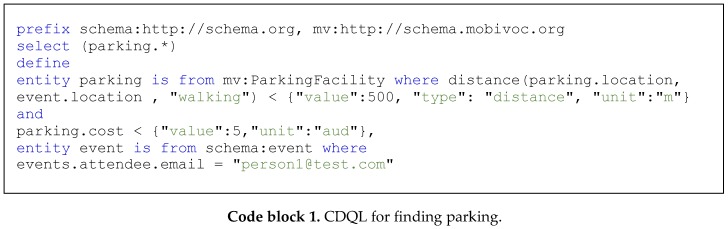



As mentioned earlier, a CDQL query also has two optional clauses, namely WHEN and CALLBACK. The WHEN clause provides support for event-based and periodic queries. Using this clause, a context consumer can subscribe to a specific situation, and the result of the query will be sent back to the consumer asynchronously when the defined situation is detected. We refer to such type of queries as PUSH-based queries. On the contrary, a PULL-based query does not contain a WHEN clauses, as it is executed only once immediately after the query has been received. 

In CDQL, in order to represent situations, we designed a specific syntax that supports rule-based reasoning, uncertainty handling, temporal relations, and windowing functionality. In Code block 2, an example of Situation representation is provided. This example defines a probabilistic ‘goodForWalking’ situation-function, which computes the probability of the comfortable walking condition for a particular location. The representation of the situation contains definitions of ranges of values for every attribute. Each attribute is assigned with a weight and, each range is assigned with a belief. The representation is based on the Context Spaces Theory (CST), and more details can be found in [30,31].

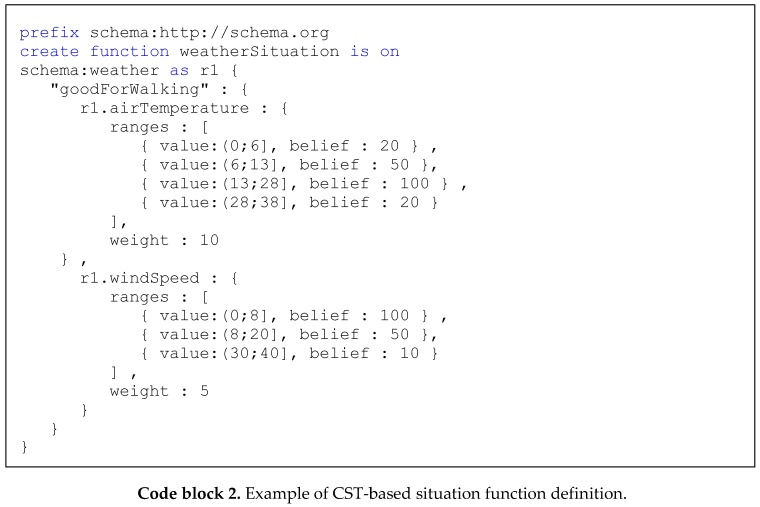



The last clause of CDQL is the SET clause, which consists of three elements, namely CALLBACK, META, and OUTPUT. The CALLBACK clause identifies how the result of queries should be sent back to the context consumers. This clause describes the callback method (e.g., HTTP Post) and other required fields (e.g., Callback URL and headers). The CALLBACK clause can be used for both push-based and pull-based queries. In the case of pull-based queries, it will allow context consumers to issue non-blocking queries and receive the result as soon as the execution of a query is finished. Regarding push-based queries, when the callback clause is presented, the result of the query will be pushed back into the subscribed entity as soon as the related situation is detected. The code snippet in Code block 3 shows an example of a PUSH-based CDQL query. This query will instruct the CoaaS platform to monitor specific parking that a car is driving to. If CoaaS detects that the carpark will be full by the time a car will arrive there, the platform suggests alternative carparks. It is worth mentioning, in order to select a list of alternative car parks, CoaaS takes the distance and walking conditions between the destination and parking into consideration by using the goodForWalking function.

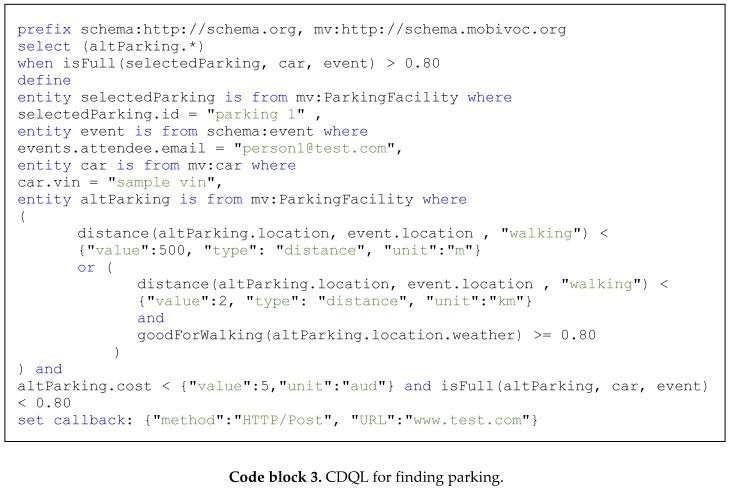



The META clause enables another essential requirement for a context query language, which is expressing different aspects of context, such as imperfectness, uncertainty, QoC, and CoC. In other words, this clause allows users to set the minimum acceptable (or default) value for each metadata. Lastly, CQL allows developers of context query to define their preferred structure of output through the OUTPUT clause. The OUTPUT clause consists of two main elements, a STRUCTURE that identifies the output data structure (e.g., XML, JSON, or ODF), and a vocabulary that specifies which semantic vocabulary should be used for each context-entity. 

In this section, we have provided an overview of CoaaS platform and presented its blueprint architecture. Moreover, we have identified the main components of CoaaS and explained their roles. As discussed in Section 2, existing IoT Context Management Platforms (CMPs) have limited support for the queries of a comparable complexity for which there is a growing demand from the industry. To address this need, we propose an efficient engine that enables execution of complex context queries in the IoT ecosystem. This engine, which is referred to as Context Query Engine (CQE), will be discussed in the rest of this paper.

## 4. Context Query Engine

**Figure 4:**
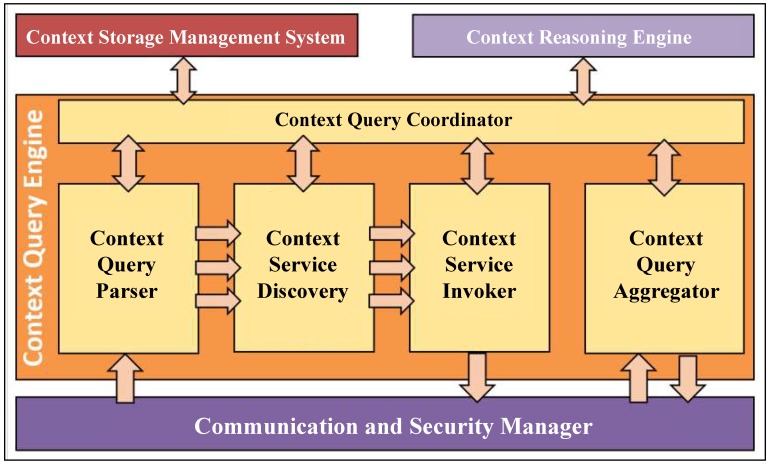
Context Query Engine Architecture.

The architecture of Context Query Engine (CQE) is illustrated in Figure 4. As mentioned earlier, this module is mainly responsible for parsing the incoming queries, generating query execution plans, orchestrating the execution of queries, and producing the final query result. Furthermore, CQE also takes care of fetching the required data from context providers on demand. 

As shown in Figure 4, there are five main components within CQE, namely (i) Context Query parser (CQP), (ii) Context Query Coordinator (CQC), (iii) Context Service Discovery (CSD), (iv) Context Service Invoker (CSI), and (v) Context Query Aggregator (CQA). A detailed description of each of these components will be presented in the remainder of this section. 

When a query is issued to CoaaS, after passing the security checks, it will be sent to the Context Query Parser (CQP) by Communication and Security Manager. The CQP has three main responsibilities, namely, parse the incoming queries, break them into several sub-queries (i.e., context requests), and determine the query’s execution plan. The details of generating the execution plan for CDQL queries are discussed in Section 4.1.

Then, the parsed query plus the execution plan will be sent to the Context Query Coordinator (CQC). The CQC plays an orchestration role in the engine. This module is responsible for managing and monitoring the whole execution procedure of a context query. We will describe the details and workflow of these components in Section 4.2.

In the next step, context requests will be pushed into the Context Service Discovery (CSD) module. This module is in charge of finding the most appropriate context service for an incoming request. The workflow of this component consists of two parts. First, it finds context services that match the requirements of a context request by utilising CSMS. Then, based on the discovered services, it returns a sorted set of the best available context services that can satisfy the requirements of a request, considering different metrics such as Cost of Service, and Quality of Service. The underlying concepts of CSD are presented in [6]. 

After selecting the best eligible context providers (i.e., context service) for each context request, the request will be passed to the Context Service Invoker (CSI). This component is responsible for fetching context from the corresponding context providers to retrieve the required contextual information and pass the retrieved information to the Context Query Aggregator (CQA). Finally, the CQA combines the results of all the context requests and generates the final result of the query. The retrieved context may also be processed by the Context Reasoning Engine (CRE) to produce high-level context. 

### 4.1. Context Query Parser and Execution Plan Generation

As stated before, CDQL supports complex context queries concerning various entities where the information about each entity might be provided by a different context service. In other words, CDQL queries are capable of expressing requests for contextual information related to one or several entities. Furthermore, entities used in a query can be dependent, which means the information retrieved from one entity might be used in the query definition of another entity. 

For example, consider the CDQL query shown in Code block 4. This query consists of three context entities, namely *vehicleA*, *trafficElements*, and *targetCarparks*, and presents a request to find all the traffic incidents that might affect *vehicleA* and also available parking options near its destination. 

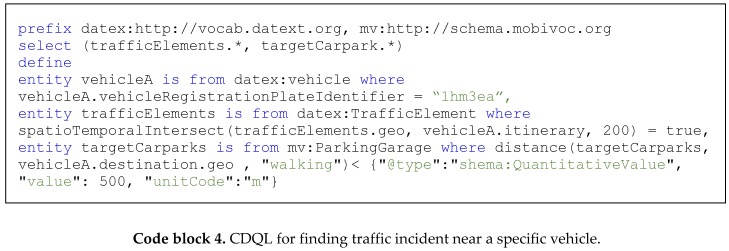



As this query shows, the definition of both *trafficElements* and *targetCarparks* are dependent on *vehicleA*, as their *WHERE* clauses have a reference to one of *vehicleA*’s attributes, i.e., *vehicleA.itinerary* and *vehicleA.destination* respectively. Consequently, before querying the registered context providers about traffic incidents and parking facilities, it is necessary to send a request to *vehicleA* for fetching its planned route (i.e., itinerary) and destination. 

On the other hand, each CDQL query might have some entities that can be queried simultaneously, which leads to reducing the overall query execution time. For example, in the query above, after retrieving the required context about *vehicleA*, both traffic incidents and parking facilities can be queried at the same time.

Based on the concepts discussed above, we have designed and developed an algorithm to generate execution plans for CDQL queries. The execution plan generation can be modelled as a graph traversal problem, by converting CDQL queries to a directed graph, where each node represents one entity, and each edge between two nodes represents the relationship (dependency) among those entities. As a result, the execution plan can be generated by finding a path that visits all the nodes in the graph, starting from a node with no dependencies (zero inbound degrees). 

**Figure 5:**
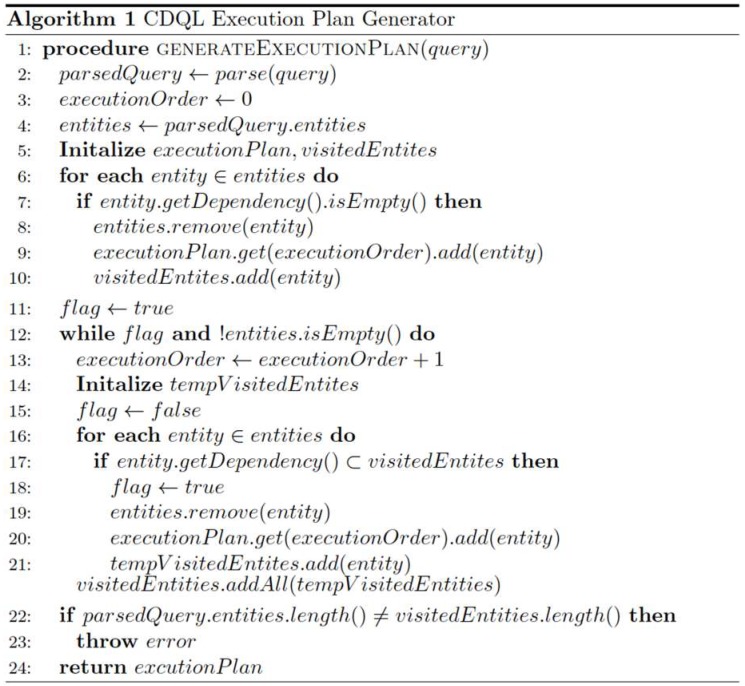
CDQL Execution Plan Generator.

The algorithm for the proposed execution plan generator is presented in Figure 5. This algorithm accepts a CDQL query and generates an execution plan that specifies the order of retrieving contextual information about the entities defined in the query. 

As the first step towards producing the execution plan, the incoming CDQL query will be parsed into an object model containing several attributes, namely *queryType*, *nameSpaces*, *select*, and *define*. The *queryType* identifies the type of the incoming query, which can be either pull-based or push-based. The *nameSpaces* element contains all the semantic vocabularies defined in the *PREFIX* clause. The *select* denotes the structure of the query’s output and includes the entities, attributes, and functions that are defined by the *SELECT* clause of the incoming query. Lastly, the *define* element is an array of context entities described in the *DEFINE* clause of the incoming query. 

Each context entity itself is represented by five elements:

- *entityID* denotes the unique name (e.g., *vehicleA*)assigned to the entity.
- *type* represents the semantic category class the entity belongs to.
- *dependency* captures the dependency with the other context entities that are referenced in the definition of this entity
- *RPNCondition* is the Reverse Polish Notation (RPN) representation of the *WHERE* clause. RPN is a well-known method for the expression notification in a postfix manner, instead of using the usual infix notation.
- *contextAttributes* consists of an array of context attributes that are used in the CDQL query in the *SELECT*, *WHEN*, or *WHERE* clauses.

Code block 5 shows the JSON representation of the parsed CDQL object for the query presented in Code block 4.

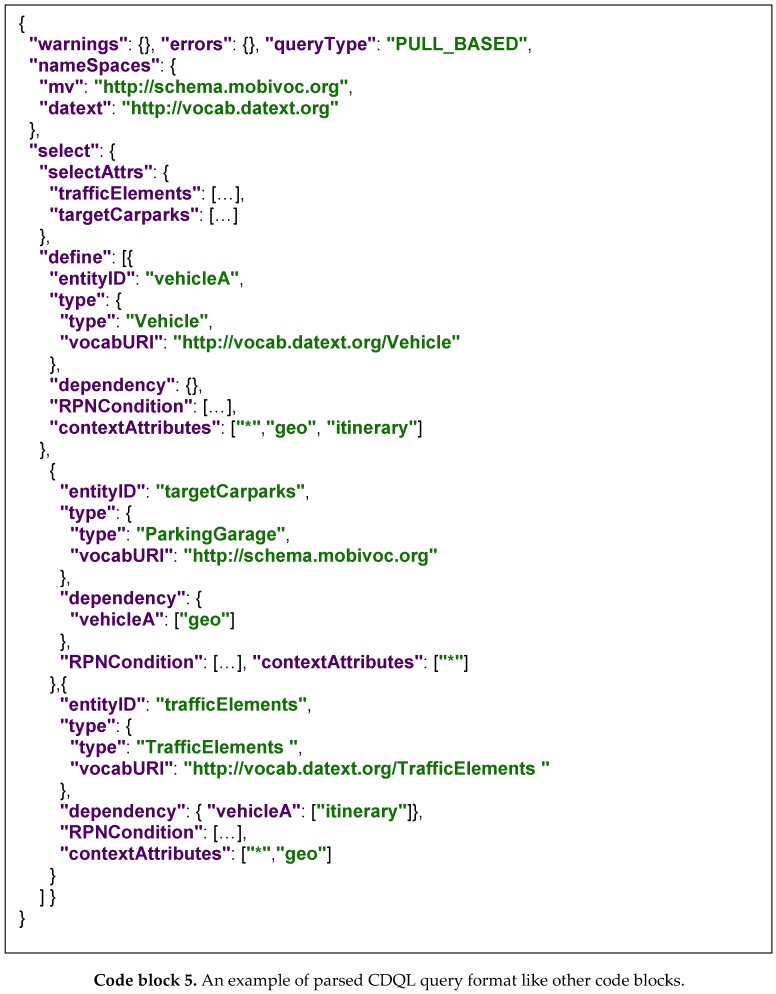



After generation of the parsed CDQL object, the initialization step of Algorithm 4.1 (Figure 5) creates an empty hashmap for storing the execution plan (*executionPlan*), and an empty set to keep track of visited context entities (i.e., *visitedNodes*). Then, the algorithm iterates over all the context entities in the *define* element to find those context entities that have no dependency (0 inbound degree). The retrieved entities in this step will be marked as visited, removed from the *define* element and will be added to the *executionPlan,* where the execution order is 1. 

As the next step, the algorithm iterates through the remaining entities in the *define* element and tries to find the entities that their *dependency* is a subset of the *visitedNodes*. Then, similar to the previous step, the found entities will be removed from the *define* element, labelled as visited, and will be added in the next execution order of the *executionPlan*. This step will be repeated several times until either all the nodes in the *define* elements are visited (until the define element becomes empty) or cannot visit a new entity in an iteration. Finally, the algorithm checks if all the entities in the *define* element are visited. If not, it means the execution plan for the incoming query cannot be generated due to a cycle in the dependency graph. Otherwise, the algorithm returns the generated execution plan.

To illustrate the procedure of generating an execution plan, consider the context query shown in Code block 6, which is an extended version of the query discussed earlier in this section in Code block 4. This query consists of four entities: *vehicleA*, *weatherCondition*, *trafficElements*, and *targetCarparks*.

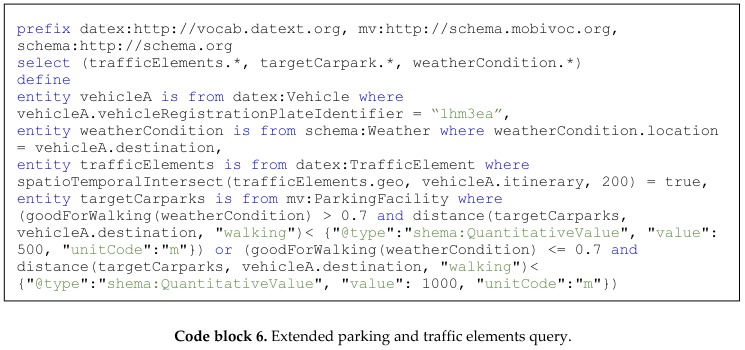



**Figure 6:**
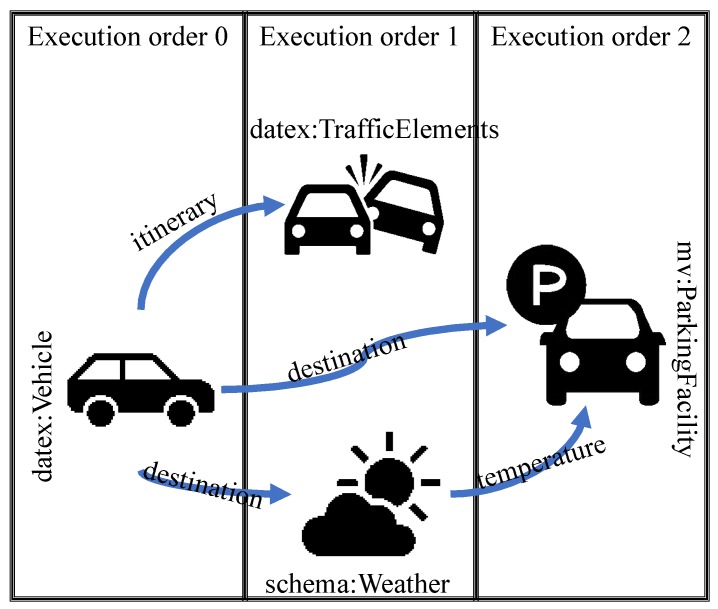
Query execution plan graph.

Figure 6 shows the directed graph for this query. As depicted in this graph, the inbound degree of entity *vehicleA* is 0. Therefore, this entity should be retrieved in the first step. In the next step, when the required information (i.e., destination and itinerary) regarding *vehicleA* is fetched, the context request related to *weatherCondition* can be issued. In the same manner, in parallel with the previous step, the request for *trafficElements* can be executed. Lastly, when the required contextual information related to *weatherCondition* is fetched, a context request will be generated to find the best available car parks. Therefore, the order of context requests execution (execution plan) for this query can be written as shown in Table 2.

### 4.2. Context Query Execution

In the previous section, we presented our proposed algorithm for the CDQL query execution plan generation. Furthermore, we showed the structure of the parsed query object and described its main elements. As the next step towards executing CDQL queries, in this section, we will describe the workflow of Context Query Coordinator (CQC) module. As discussed in Section 3.2, CQC is responsible for managing the whole execution lifecycle of CDQL queries.

As mentioned in Section 3.3, CDQL supports querying contextual information using two approaches: the pull-based approach and the push-based approach. In the remainder of this section, we will discuss how CQC handles pull-based queries. The execution of push-based queries falls out of the scope of this paper. 

By default, Pull-based CDQL queries are executed synchronously. A synchronous query is a query that maintains control over the process of the application that issues the query for the query’s lifetime. In other words, when a context consumer issues a pull-based query, it has to wait for the entire round trip, from when the query is first sent to the CoaaS until the results are retrieved and returned to the context consumer. However, if the CALLBACK method is presented in a Pull-based query, the query will be executed in a non-blocking fashion, and the outcome of the query execution will be pushed to the corresponding context consumer. 

**Figure 7:**
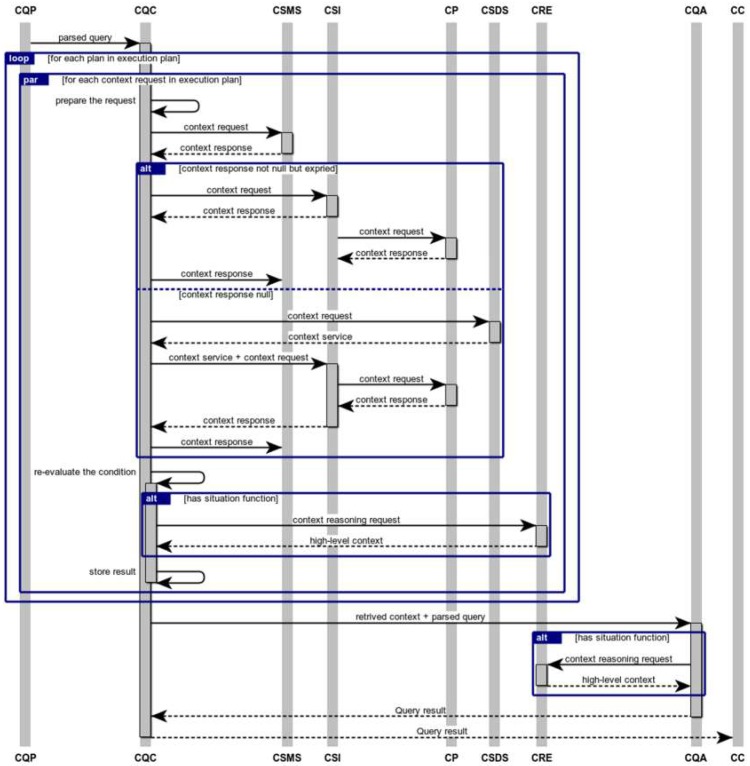
CDQL execution workflow.

The complete workflow of executing pull-based queries is illustrated as a flow of events in a sequence diagram in Figure 7. When CQE receives a CDQL query, the query will be sent to CQP, which parses the raw query and generates the execution plan. Then, the CQP passes the parsed query object plus the execution plan to CQC. As described in Section 4.1, each execution plan consists of several execution orders that specify the correct sequence of retrieving the context entities defined in a CDQL query. Moreover, each execution order itself has one or several independent entities, which means they can be queried simultaneously. 

Therefore, to execute an incoming context query, CQC iterates over the generated execution plan in ascending order, from the execution order 1 to the last execution order. Following this, for each entity in the current execution order, CQC starts a new thread that forms and issues a context request to fetch the required context of the entity. As defined in Definition 6, context requests are represented as a triple: 〈E, CA, P〉 where E denotes the type of entity of interest (i.e., *entityType* in the parsed query object), CA is a set of requested context attributes (i.e., *contextAttributes* in the parsed query object), and P. is a set of predicates, which are defined over *CA* using logical expressions (i.e., *RPNCondition* in the parsed query object). Execution of context requests has four main steps, as outlined below:

Step 1: The generated context requests will be initially sent to the Context Storage Management System (CSMS). CSMS searches the repository of registered entities by converting the incoming context requests to the underlying data storage language. Subsequently, a list of matching context entities (i.e., context responses) will be sent back to the CQC, which can have zero or more entities, depending on the ability of CSMS to find compliant entities. 

Step 2: If the returned list is non-empty, the CQC checks the validity of the context responses by inspecting the expiry timestamps of their context attributes. If any of the attributes were expired, CQC issues a request to Context Service Invoker (CSI) to re-fetch the value of the expired context attribute from the corresponding context provider. 

Step 3: On the other hand, if CSMS cannot find any context entity that matches the characteristics of the requested entity, CQC issues a context discovery request to CSD. Then, CSD tries to find and select the most eligible context services that match the requirements of the incoming context request. Details of how CSD discovers and selects matching context services is provided in [6]. Then, CQC fetches the context of the entities of interest through the CSI module. 

Step 4: In the final step of handling context requests, CQC re-evaluates the RPNCondition of those retrieved entities that their context attributes have been updated in Step 2. Moreover, if the RPNCondition contains any situation or aggregation function that cannot be evaluated in the previous steps, CQC re-evaluates them. 

After successfully obtaining the needed context for each request in the first execution order, CQC stores the result and starts the next iteration, by incrementing the execution order by one. However, before starting the next iteration, it is required to update the *WHERE* clause of those entities that are dependent on at least one of the entities that are retrieved in the current execution order. Consequently, CQC traverses the context entities in the next execution orders and updates their condition by replacing the dependant context attributes according to their actual values that are fetched in the current iteration. During the process of updating the RPNConditions, there might be a case that more than one instance of context entity is retrieved for a given context request, which is referred to in the *WHERE* clause of another entity. In this situation, if it is required, CQC reformulates the *WHERE* clause of the dependent entity. Based on how the dependent attribute is used in the *WHERE* clause, five different reformulation strategies might be considered by CQC. Table 3 shows the reformulation strategies.

Finally, when all the context entities presented in the execution plan are retrieved, the fetched context will be passed to the Context Query Aggregator (CQA). CQA generates the final output of the incoming CDQL query based on its SELECT clause. 

To further clarify the execution procedure of pull-based queries, consider the example query presented in Code block 7. This query is designed to find the vehicles that are driving faster than 60 km/h at a distance less than 500 m from any school in one of Melbourne’s suburbs. 

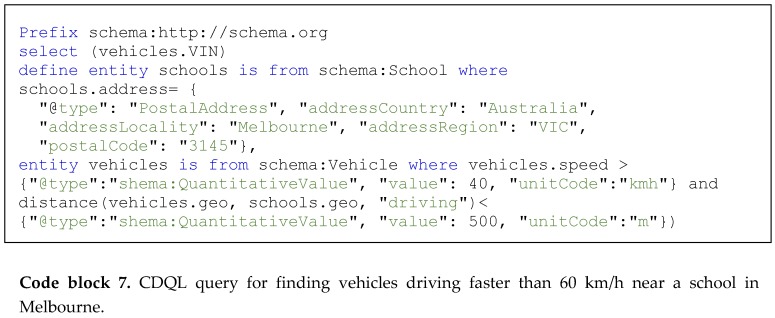



The code block shows that this query has two entities, *schools* and *vehicles*, where the *vehicles* entity has a dependency on entity *schools*. Therefore, the execution plan of the query has two execution orders that is shown in Table 4. Based on the execution plan, CQC issues a context request to CSMS to find all the schools within the specified area:

$$cr_{schools}:\;\langle schema:School,\;\left\{address,\;geo\right\},\;\left\{schools.address=\left\{\ldots \right\}\right\}\rangle$$

Then, CSMS queries the repository of the registered entities to find the matching schools. For this query, assume three schools are registered inside the identified region. Therefore, CSMS sends a context response back to CQC, which contains the address and geocoordinates of three schools that match the aforementioned condition. Then, for each of these entities, CQC validates the expiry timestamp of the corresponding context attributes. However, as both addresses and geocoordinates for an entity like a school are considered as static values, we assume all the retrieved context attributes are valid. 

Since the entity *schools* is the only entity in the first execution order, CQC starts the next execution order. However, as mentioned earlier, it is required to update the RPNCondition of the entity *vehicles* by replacing the *schools.geo* by its actual value. For the given example, as more than one entity has been found for *schools* entity, CQC reformulates the WHERE clause of entity *vehicles*. The reformulated query can be seen in Code block 8. 

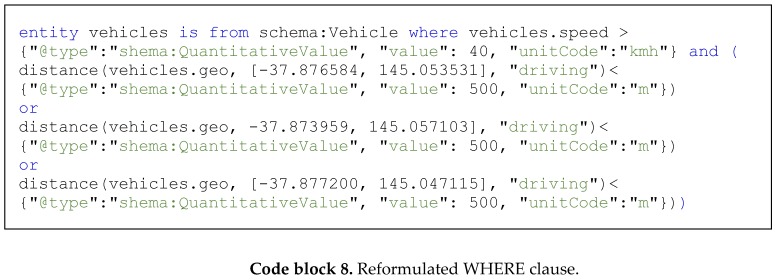



In the next step, CQC forms a context request based on the updated RPNCondition in order to find the vehicles that are over-speeding near one of the three schools found in the previous iteration:

$$cr_{\mathrm{vehicles}\;}:\;\langle schema:\mathrm{Vehicle},\;\left\{VIN,\;geo,speed\right\},\;\{vehicles.distance<\ldots \}\rangle$$

This time we assume CSMS returns 10 vehicles that each of them meets the above conditions (i.e., near the school and over-speeding). Then, for each vehicle, CQC checks the expiry date of their required attributes, namely Vehicle Identification Number (VIN), geocoordinate, and speed. As both speed and geo coordinate for a mobile entity like a vehicle have high update frequency, there is a considerable chance of having outdated values. If the values are expired, CSI sends a request to corresponding vehicles to fetch the real-time values of the expired context attributes. Then, finally, CQC re-evaluates the RPNCondition based on the updated context attributes and returns the VIN of over-speeding cars back to the corresponding context consumer. 

## 5. CQE Implementation

**Figure 8:**
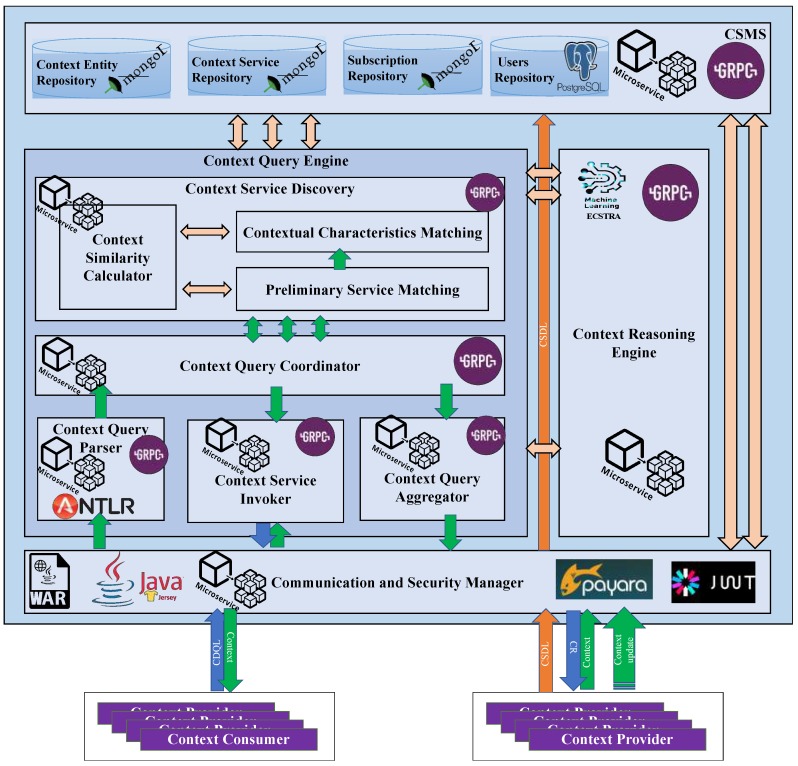
Architecture of the CoaaS platform prototype implementation.

Based on the reference architecture of Context-as-a-Service platform and the concepts presented in the previous sections, we have implemented a prototype of the CoaaS platform. Figure 8 presents the architecture of the implemented context management platform.

As described in Section 3.2 the CoaaS platform consists of five main components: (i) Communication and Security Manager (CASM), (ii) Context Query Engine (CQE), (iii) Situation Monitoring Engine (SME), (iv) Context Storage Management System (CSMS), and (v) Context Reasoning Engine (CRE). In the current implementation, which has around 1.3 million lines of code, we have developed CoaaS as a Microservice-based application using Java Enterprise Edition 7 (Java EE 7) framework. In this regard, each of the abovementioned components is implemented as a separate microservice. Therefore, the implemented prototype of CoaaS platform is very scalable. The rest of this section briefly presents the description of the implementation of each of these components. 

The *Communication and Security Manager (CASM)* is implemented as a RESTful web service using Jersey 2.8 framework (https://jersey.github.io/). CASM provides an interface that supports the proposed languages presented in [11], namely Context Definition and Query language (CDQL) and Context Service Description Language (CSDL) and. Using this interface, clients can perform several operations, such as querying contextual information, registering context services, updating context information, and subscribing to certain situations about their entities of interest. 

**Figure 9:**
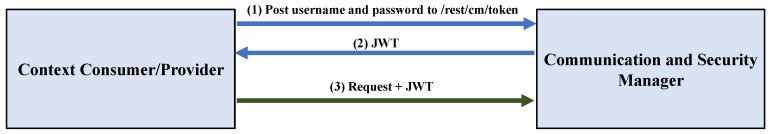
Authentication and authorisation mechanism.

Moreover, this component is enhanced with a token-based authentication and authorisation mechanism, which is implemented through JSON Web Token (JWT) (https://jwt.io/). JWT is an open standard that defines a compact and self-contained way for securely transmitting information [32]. The diagram depicted in Figure 9 shows how the implemented authentication and authorisation mechanism works. 

In the first step, clients acquire an authorisation token by sending an authentication request that contains the client’s username and password to the CASM via the URL “/rest/cm/token”. Then, based on the provided credentials, CASM authenticates the user by using Java Authentication and Authorization Service (JAAS) (https://docs.oracle.com/javase/8/docs/technotes/guides/security/jaas/JAASRefGuide.html). If the client is successfully authenticated, a JSON Web Token (JWT) will be returned. Code block 9 shows how JWT can be acquired:

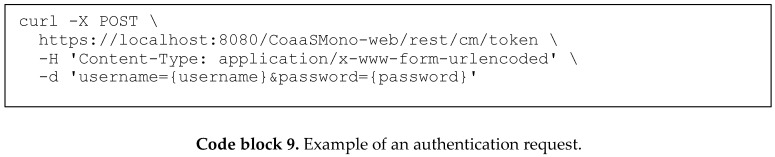



Using the acquired token, clients can securely invoke CoaaS APIs. In this regard, they should provide the JWT in the *‘Authorisation’* header of the HTTP request using the *‘Bearer’* schema. Then, CASM checks for a valid JWT in the *‘Authorisation’* header, and if it is present, the client will be allowed to access protected resources.

The CoaaS platform has four main Restful APIs that are presented in Table 5. To enable secure communication between clients and CoaaS, all these APIs are only accessible via HTTPS protocol. 

The main API for context consumers is the CDQL query interface, which is accessible via the URL ‘/rest/cm/token’. This interface accepts a CDQL query as input; based on the type of the provided query, it returns either contextual information or status of the executed query (in the case of CDL queries, e.g., create function). The code snippet provided in Code block 10 shows how this interface can be invoked:

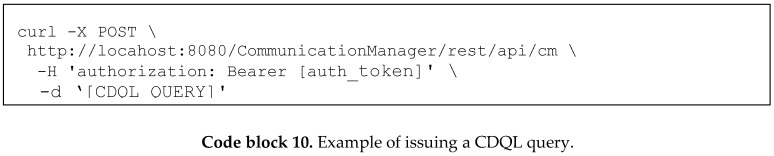



As explained in Section 3.1, CoaaS can interact with context providers (CP) in two ways, either by fetching context on-demand or through receiving context/data streams. In the first case, the CPs must have registered the description of their services by sending a context service registration request. In order to do this, they need to describe their context service using CSDL language and send the service description as a body of an HTTP POST request to the CoaaS service registration API (i.e., /rest/cm/register/). After successfully registering a context service, CoaaS can retrieve data about the registered service’s IoT entities by sending requests to the corresponding provider on-demand. 

As mentioned above, CoaaS can also process streams of context updates, which CPs are sending to the platform. Context updates contain updates of the entities’ states and are processed by CoaaS to monitor situations. Therefore, CoaaS has an API that allows CPs to send context updates to the CoaaS platform. These updates are cached in the CoaaS storage (i.e., CSMS), mainly for the purpose of using these data to serve pull-based queries. The code snippet provided in Code block 11 shows how the context update API can be invoked.

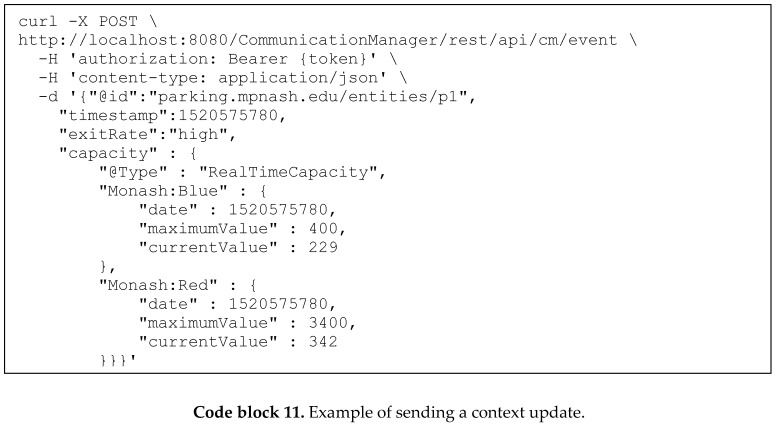



The *Context Query Engine (CQE)* has been implemented as a gRPC server (https://grpc.io/), based on the provided architecture in Section 4 and the concepts and the algorithms presented in preceding sections. To parse the incoming queries, a query parser is developed by using Antlr 4.6 (https://www.antlr.org/). ANother Tool for Language Recognition (ANTLR) is a parser generator for reading and processing structured text. This framework accepts a formal grammar (written in an EBNF like format) as input and generates a parser for that language. The generated parser can automatically build parse trees, which are data structures representing how a grammar matches the input. ANTLR also automatically generates tree walkers that can be used to visit the nodes of those trees to execute application-specific code. 

To implement the *Context Reasoning Engine (CRE)*, we have adopted an existing context-awareness and situation-awareness framework called ECSTRA [33]. ECSTRA builds on the basis of context spaces theory [30]. This framework provides a comprehensive solution to reason about the context from the level of sensor data to the high-level situation. In order to integrate ECSTRA in CoaaS, we have implemented another microservice that uses a Java implementation of ECSTRA framework. Using this microservice, other components can use the ECSTRA framework to reason about context data. 

The *Context Storage and Management System (CSMS)* has been implemented based on the architecture presented by [28]. In the current implementation, CSMS has five main modules, namely context repository, context service discovery repository, context history repository, subscription repository, and user management database. The first four repositories, which are used to store context data, have been implemented using MongoDB (https://www.mongodb.com/). On the other hand, the user repository that contains clients’ profile, including their credentials, is implemented as a relational database using PostgreSQL (https://www.postgresql.org/). Moreover, CSMS has an interface, which is also implemented as a gRPC server that allows other components to access and store data in the aforementioned repositories.

**Figure 10:**
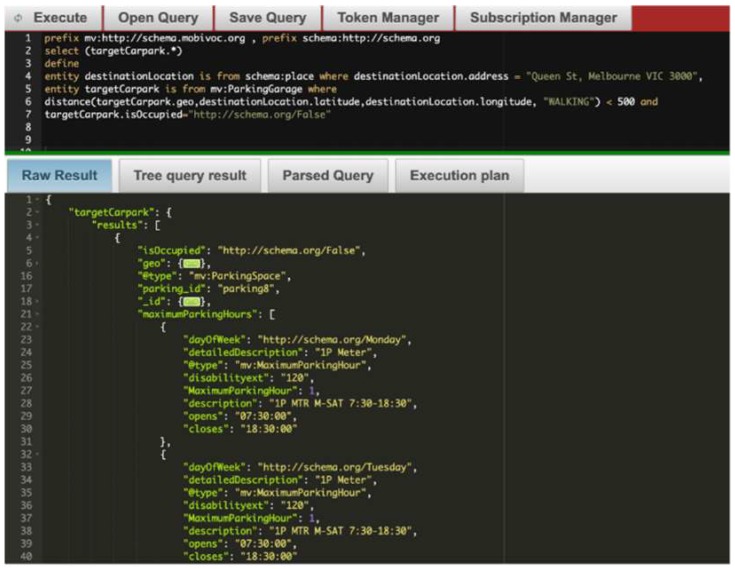
CoaaS IDE.

Furthermore, to ease the development of context queries and service definitions, a specialised web-based IDE has been developed. The main features of the IDE are: (i) CDQL syntax highlighting, (ii) auto-completion of CDQL keywords and terms coming from integrated semantic vocabularies and standards, (iii) visualising the execution plan of parsed query, (iv) showing errors, warnings, and recommendations to CDQL developers, and (v) managing authorization tokens. A screen dump of the CoaaS IDE is presented in Figure 10. 

The current implementation of CoaaS platform is available as several Docker (https://www.docker.com/) images and can be downloaded from the following link: https://hub.docker.com/r/coaas.

## 6. Evaluation of CQE

This section describes the performance evaluation of the proposed Context Query Engine (CQE) that provides support for the execution of complex context queries represented in CDQL. In order to show the merits of the proposed engine, we compared the performance of our approach with Fiware Orion CMP [34] in the execution of different context queries. In the rest of this section, we will first describe the experimental environment and metrics of our evaluation. Then, based on the provided metrics, we will present several experiments to evaluate the performance of the proposed CQE during the execution of basic and complex context queries. 

### 6.1. CQE Experiment Environment

In order to evaluate the proposed solution, we used the current implementation of the CoaaS platform, which was described in detail in Section 5. During all the conducted experiments, the CoaaS platform was running as a docker container. The docker container is hosted on a virtual machine located in the Deakin University Cloud and running Debian GNU/Linux 8 (Jessie). The VM is running on a four-core Intel(R) Xeon(R) CPU E5-2660 v3 @ 2.60 GHz instance with 16 GB RAM. Moreover, for the deployment of CoaaS platform, we have created a docker-compose file that sets up all the required development environment and automates the installation and configuration process. The docker-compose file is available online at https://github.com/ahas36/Execution-of-Complex-Context-Queries-in-Dynamic-IoT-Environments/tree/master/CoaaS.

In a similar manner, the Fiware Orion context broker was also deployed as a docker container on another virtual machine (with exact same specification) hosted on Deakin University Cloud. To deploy the Fiware Orion context broker, we used the docker-compose file provided in Fiware GitHub (https://github.com/telefonicaid/fiware-orion/blob/master/docker/docker-compose.yml. Moreover, to establish a fair comparison, we carefully tuned the Orion context broker by modifying the docker-compose file based on the official performance tuning guide (https://fiware-orion.readthedocs.io/en/master/admin/perf_tuning/index.html presented in the Fiware documentation. The modified docker-compose file is available online at https://github.com/ahas36/Execution-of-Complex-Context-Queries-in-Dynamic-IoT-Environments/tree/master/Orion.

For our experiments, we have developed a Java application for simulating context entities. This application offers a method that accepts the structure of an entity (a string that represents the entity type and a key-value map that represents a collection of context attributes name and their value’s type) and the number of instances of the entity that needs to be generated. Then, the entity simulator application randomly generates context entities based on the provided structure and registers them in both CoaaS and Orion platforms. Moreover, this method can generate multiple dependent context entities, where the value of an attribute of one entity is equal to the context attribute’s value of another entity. The source code of the entity simulator application is available at https://github.com/ahas36/Execution-of-Complex-Context-Queries-in-Dynamic-IoT-Environments/tree/master/EntitySimulator.

For all the experiments in this section, we used JMeter 4 to generate and issue CDQL and NGSI queries. We deployed the JMeter 4 in the same network where the CoaaS and Orion instances were running in order to minimise the network delay. The JMeter test plans for all the conducted experiments are available online at https://github.com/ahas36/Execution-of-Complex-Context-Queries-in-Dynamic-IoT-Environments/blob/master/Experiments/testplan.jmx. 

### 6.2. CQE Experiments Metrics

To measure the performance of the proposed solution, an evaluation framework is required. In this paper, based on the benchmarking approach presented in [13], we considered two main metrics that can affect query execution performance. These metrics are: (i) the number of parallel queries, and (ii) the richness of incoming queries. The following list provides a brief overview of each of these metrics and explains how they can impact the query execution’s performance:

- *The number of parallel queries*: The increase in the number of parallel queries can lead to performance degradation due to the resource limitation.
- *The richness of a query*: The structure of each context query might also influence the execution performance. The main features that can impact the query execution performance are the number of defined entities, the number of constraints, the ratio of ‘ANDs’ to ‘ORs’, the number of functions, and the type of functions.

In the rest of this section, we will design several experiments based on the metrics discussed in this section to measure the performance of CoaaS and Orion platform in the execution of context queries.

### 6.3. CQE Experiments and Results

In this section, we describe a set of experiments which has been designed to evaluate the performance of CoaaS and Orion platforms in the execution of context queries. At first, we evaluated the performance of the execution of basic context queries, where each query only contains one entity type. To this end, we studied the impact of the query load on query execution performance to show how CoaaS and Orion platforms perform when the number of parallel queries increases. In order to achieve this goal, we gradually increased the query load (query per second) from 100 QPS to 1000 QPS and measured the average query response time and achieved throughput. Moreover, to take the impact of the richness of queries into account, we repeated this experiment three times using different queries with different levels of richness. 

The first query (Q1) represents the most basic form of a context query that only contains one equality constraint (i.e., *attribute = “value”*). In the second query (Q2), we extended the previous query by adding an additional inequality constraint (i.e., attribute < number) connected with ‘AND’ operator. The third query (Q3) is a location-based query, which searches for all the instances of an entity of a specific type near a random coordinate. To make sure all the queries are returning a roughly similar number of entity instances in the response, we have specified the maximum number of returning instances to 10 for all the queries. These three queries designed in a way that they can be represented in both CDQL and NGSI v2 languages. Hence, in these experiments, we did not consider some of the advanced features of CDQL queries, such as using *‘OR’* operator, situation functions, or custom aggregation functions, as they are not supported in NGSI v2.

In the first set of experiments, we have used our entity simulator application to generate 2000 instances of an entity with type “Entity1”, which has four context attributes. Table 6 shows the details of these context attributes.

**Figure 11:**
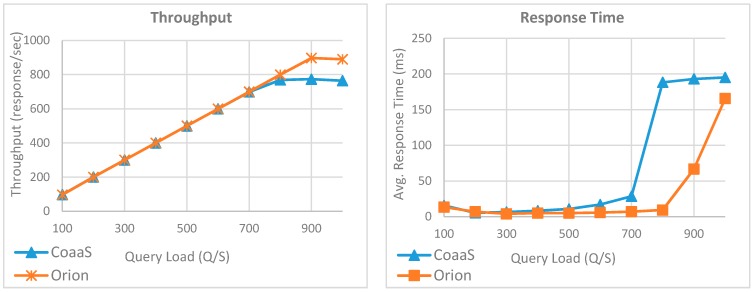
Impact of increasing number of parallel queries for Q1.

The result of this experiment for the first query (i.e., Q1) is presented in Figure 11. The result shows, the CoaaS platform was able to handle up to 700 QPS, where the average query response time was less than 28 ms. In the case of Orion, we can observe that the performance was slightly better in the execution of Q1. Orion was able to serve 800 QPS without a considerable impact on the query execution time. However, the result of this experiments shows that the performance of both platforms degraded when the query loads became more than the maximum achievable throughput of each system, which leads to a dramatic increase in the average query response time. To conclude, we can say that Orion slightly outperformed CoaaS in the execution of Q1, where the maximum achieved throughput of CoaaS and Orion were 799 and 897 response per second, respectively.

**Figure 12:**
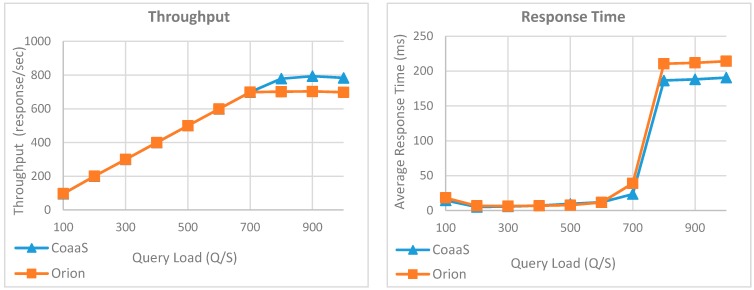
Impact of increasing number of parallel queries for Q2.

Figure 12 compares the performance of CoaaS with Orion platform in the execution of the second query (i.e., Q2). The result shows that the performance of CoaaS platform remained almost unchanged in comparison with the execution of the first query. We can observe that CoaaS was able to handle up to 700 queries per second, where the average query response time was less than 24 ms. 

In the case of Orion, we can witness a slight performance drop compared to Q1, where the maximum achieved throughput reduced from 897 response per second to 702 response per second. Similar to the previous experiment, we can observe a sharp increase in the average response time for both platforms when the query loads exceed 700 QPS. To sum up, based on the outcome of this experiment, we can see the performance of CoaaS in executing the second query is better than Orion. Hence, it can be inferred that CoaaS’ performance is more resistant to the increase in the complexity of queries, compared to Orion.

**Figure 13:**
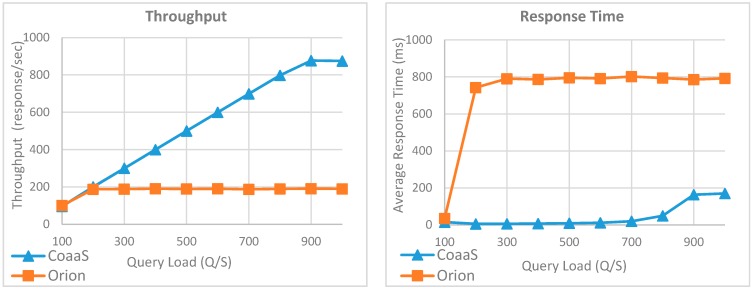
Impact of increasing number of parallel queries for Q3.

Figure 13 illustrates the outcome of the third iteration of the first experiment, where we measured the performance of CoaaS and Orion in the execution of the third query (i.e., Q3), which is a geolocation-based query. As can be seen in this figure, unlike the first two experiments, there is a big gap between the performance of CoaaS and Orion in terms of both achieved throughput and average response time. The results show CoaaS has a better overall performance in the execution of Q3 compared to its own performance in the execution of the first two queries. The reason behind this observation is CoaaS automatically indexes the context attributes with geo coordinates values. Therefore, we can see the achieved throughput of CoaaS reached 876 response per second where the query load was 900 Q/S. On top of that, the response time graph shows the maximum average response time for Q3 was 169 ms, where the query load was 1000 Q/S. In contrast, the results reveal a big drop in performance of Orion during the execution of Q3. As can be seen in the graph, increasing the number of parallel queries to more than 100 Q/S leads to a dramatic growth in average response time of queries, where the value went from 35 ms to 742 ms. Moreover, the throughput graph shows the maximum achieved thought of Orion is 189 response per second, which is 4.6 times less than CoaaS achieved throughput. 

So far, we have discussed the performance evaluation of CoaaS and Orion platforms in terms of executing context queries concerning a single entity type and showed that CoaaS provides better overall performance when the richness of queries increases. As mentioned in Section 6.2, another important factor that can increase the query richness is the number of entities involved in the query. Therefore, in the rest of this section, we will focus on the impact of the number of entities on query execution performance. To this end, we have designed two experiments. 

In the first experiment, we conducted a test similar to the last three experiments, but this time we used a new query that contains two entity types. Code block 12 presents the CDQL query template for this test, where {N} and {M} will be replaced with two randomly generated numbers. 

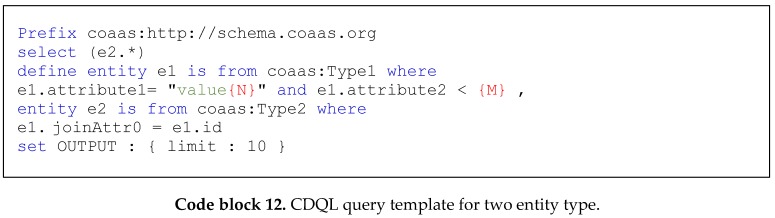



As mentioned earlier, NGSI v2 does not support having more than one entity types per query. As a result, in order to implement a similar query showed in Code block 12 using NGSI v2, it is required to issue two sequential NGSI queries. The first query fetches the ID of up to 10 instances of entity Type1 that match the search criteria. The second query searches for the entities of Type2 where the value of their *joinAttr0* is equal to one of the IDs retrieved in the first query. 

For this experiment, we used our entity simulator software to generate and register two types of entities, Type1 and Type2, where the number of generated instances for each type was set to 1000. We have used the same structure for context attributes of entity Type1, as what we used in the first set of experiments. The used structure for context attributes of entity Type2 is shown in Table 7.

**Figure 14:**
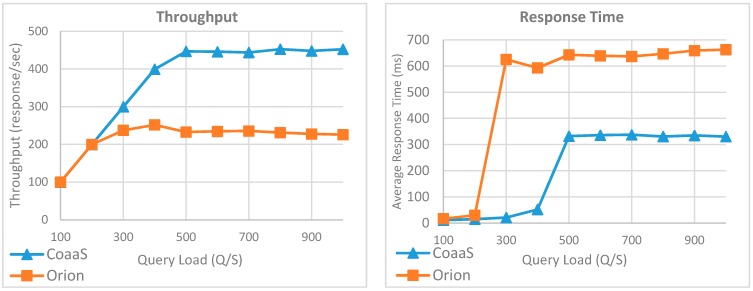
Impact of increasing the number of parallel queries for a query with two entity types.

The result of this experiment is represented in Figure 14. As the result shows, the performance of CoaaS and Orion was almost identical when the query load was less than 200 QPS. However, when the query load became larger than 200 QPS, a drastic performance degradation in Orion can be observed. In contrast, CoaaS was able to serve the context queries without any considerable impact on the average response time while the query load was less than 500 QPS. 

Moreover, we can observe that the maximum achieved throughput of CoaaS is 446 responses per second, which is almost two times better than what Orion could achieve. On top of that, based on the presented results, the average response time of CoaaS was less than half of the Orion average response time when the query load exceeded 500 QPS. To conclude, on the basis of this experiment, we can say the overall performance of CoaaS in the execution of a query with two entities is almost two times better than Orion. 

To further study the impact of the number of entities on execution performance, we have conducted another experiment. In this experiment, we fixed the query load to 200 QPS. The reason that why we choose this value was both CoaaS and Orion was able to serve the query in the last experiment with decent performance when the load was 200 QPS. Then, we gradually increased the number of entities in queries from 1 to 10 and measured the performance of both CoaaS and Orion platforms in terms of achieved throughput, average response time, and average Bytes sent/received for each query. It worth mentioning that unlike the previous experiments, we executed this experiment from a computer outside the Deakin University network, where the CoaaS and Orion platforms where running. The reason behind this decision was as the number of issued queries for CoaaS and Orion were different in this experiment, we wanted to take the possible network latency into account. To generate and register the context entities for this test, we have followed a similar approach to the previous test. However, for this experiment, we defined 10 entities with different types, and for each entity type, randomly generate and register 1000 instances. 

**Figure 15:**
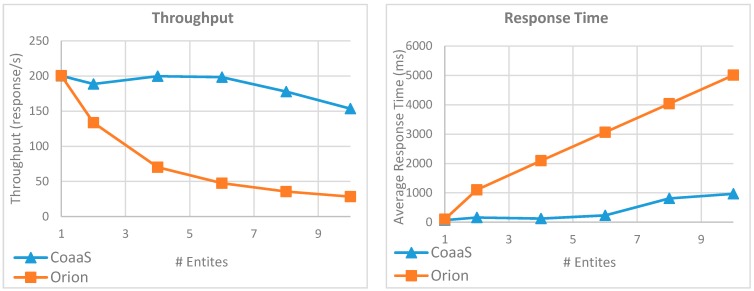
Impact of increasing the number of entities in a query.

Figure 15 presents the outcome of this experiment. As the result shows, the achieved throughput of CoaaS platform was equal to the expected throughput (i.e., 200 response/Second) and the average response time was less than 233 ms while the number of entities in a query was less than 7. However, a slight performance drop occurred when the number of entities became more than 7. As it can be seen in the result, the achieved throughput and average query response time of CoaaS platform were 153 response/second and 968 ms respectively, when the number of entities per query was 10. In contrast, the performance of the Orion platform decreased dramatically by increasing the number of entities per query. As the throughput and response time graphs show, the achieved throughput of Orion decreased from 200 to only 28 responses per second and the average response time increased from 99 ms to more than 5 s.

**Figure 16:**
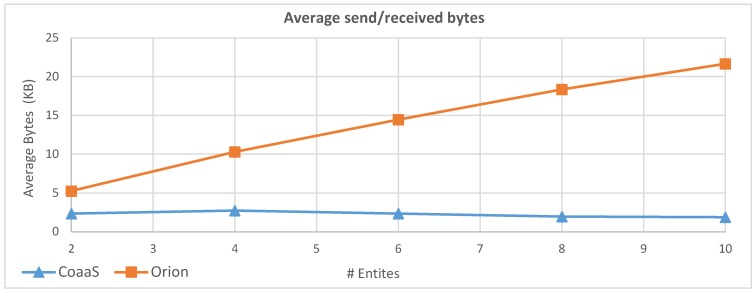
Network Load.

Figure 16 illustrates another advantage of CoaaS compared to Orion in the execution of queries with more than one entity. This image shows the average amount of Bytes sent/received for each query. As the result shows, the size of sent/received Bytes for CoaaS platform does not have any correlation with the number of entities per query, and it remained almost unchanged throughout this experiment. In contrast, in the case of Orion, we can observe a direct relationship between the number of entities per query and the amount of communicated Bytes. As the graph shows, this value was increased from 5255 to 21,654 bytes. The difference in the amount of the transferred bytes explains one of the main reasons why CoaaS outperformed Orion in the execution of complex context queries with multiple entities.

In all the conducted experiments, we demonstrated how the CoaaS platform is handling a considerable query load with high performance. Further, we showed the overall performance of the CoaaS platform is higher than the performance of the Orion platform, especially in the case of executing complex context queries. 

Moreover, as we demonstrated in our earlier publication [11], CDQL offers more features and flexibility to the developers of context-aware IoT applications, compared to NGSI. For instance, several NGSI queries are required to implement a use case, which can be implemented with only one CDQL query. Therefore, we can state that using CDQL makes the development and maintenance of context-aware IoT applications more straightforward, as fewer queries are needed to be composed to achieve the desired functionality. 

Additionally, CDQL provides several other features, such as aggregation functions, window functions, situation inference functions, and temporal relations, that are essential for a CMP but not supported in NGSI. The understanding of the necessity of this extended functionality was obtained from our collaboration with one of the biggest German car manufacturing companies [11], where we demonstrate how CoaaS platform can be utilised to provide real-time context to a smart vehicle.

To conclude, we can claim that the CoaaS platform not only has better overall performance in the execution of context queries compared to Fiware Orion, but also provides more functions to the developer of context-aware IoT applications. Based on the discussion above, we believe the proposed CMP has a high degree of industrial applicability.

## 7. Conclusions

In this paper, we have proposed, designed, implemented, and evaluated a mechanism for the execution of complex context queries in near real-time. This mechanism, which is an integral part of Context-as-a-Service platform, called Context Query Engine (CQE). The Context Query Engine (CQE) is mainly responsible for parsing the incoming queries, generating and orchestrating the query execution plan, and producing the final query result. Furthermore, CQE is also in charge of finding the most appropriate context service for an incoming request. To assess the performance and scalability of CQE in the execution of context queries under different scenarios, we conducted two sets of experiments and compared the results with the Orion context broker. The result showed that CQE and Orion context broker have similar performance in executing of basic context queries. However, the performance of CQE increased dramatically in comparison to Orion when the complexity of the queries increased (i.e., the number of entities per query become more than 3) with CQE achieving four times higher throughput than Orion. To conclude, based on the outcome of this paper, we can state that supporting complex context queries can increase the overall performance of context management platforms.

Despite the contributions of this work towards operationalising context-awareness in the IoT ecosystem, there are still several open issues in this domain that require further investigation. Two of the main interesting challenges that we are planning to tackle in our feature works are:

- *Auto-Scaling Strategy*: In this paper, we have conducted all the experiments on a single instance of CoaaS platform. However, in production environments, context management platforms are needed to be scaled-out to deal with the massive number of requests generated by the billions of IoT devices. To address this challenge, we are planning to investigate and design an auto-scaling strategy for CMPs that automatic scale-out or scale-in based on the scale of incoming requests.
- *Context Prediction*: Another important aspect of context processing is context prediction. Context prediction is referred to as the process of exploiting expected future context of IoT entities based on the historical context. A CMP that supports context predication offers distinct advantages to the context consumers, which enables a range of new use-cases. Hence, we are planning to investigate, design, and implement a generic mechanism that allows context consumers to predict the future context of IoT entities.

## Figures and Tables

**Table 1:** CoaaS major components.

Component	Responsibilities
**Communication and Security Manager**	(iv) Privacy, security and access control
**Context Query Engine**	(i) Sensor data acquisition (iii) Context service registration and discovery (vi) Context querying (Context diffusion and distribution)
**Situation Monitoring Engine**	(i) Sensor data acquisition (v) Context processing and reasoning
**Context Storage Management System**	(i) Sensor data acquisition (ii) Current and historical context storage (iii) Context service registration and discovery
**Context Reasoning Engine**	(v) Context processing and reasoning

**Table 2:** Example of execution plan

Execution Order	Entities
Execution Order 1	vehicleA
Execution Order 2	weatherCondition trafficElements
Execution Order 3	(i) Sensor data acquisition (v) Context processing and reasoning

**Table 3:** RPN condition reformulation strategies.

Usage Type	Strategy	Example *	
		Original Condition	Reformulated Condition
**In a condition using set operators (e.g., containsAny, containsAll)**	No changes required.	e_1_.a_1_ containsAll e_2_.a_1_ e_1_.a_1_ containsAny e_2_.a_1_	e_1_.a_1_ containsAll [1,2,3,4] e_1_.a_1_ containsAny [1,2,3,4]
**In an equality condition**	The equality operator will be replaced by containsAny.	e_1_.a_1_ = e_2_.a_1_	e_1_.a_1_ containsAny [1,2,3,4]
**In an inequality condition**	The inequality will be broken down into several inequality conditions (one for each instance of dependent entity) that are connected with *OR* operator.	e_1_.a_1_ < e_2_.a_1_	(e_1_.a_1_ < 1 or e_1_.a_1_ < 2 or e_1_.a_1_ < 3 or e_1_.a_1_ < 4)
**Inside a function call**	The function call will be broken down into several function calls (one for each instance of dependent entity) that are connected with *OR* operator.	F_1_ (e_1_.a_1_, e_2_.a_1_) < 12	(F_1_ (e_1_.a_1_, 1) = true or F_1_ (e_1_.a_1_, 2) = true or F_1_ (e_1_.a_1_, 3) = true or F_1_ (e_1_.a_1_, 4) = true)
**Inside an entityMatch operator**	For each instance of dependent entity, one entityMatch statement will be generated. The *OR* operator will be used to connect these statements.	entityMatch(e_1_.a_1_ = e_2_.a_1_ and e_1_.a_2_ < e_2_.a_2_)	((e_1_.a_1_ = 1 and e_1_.a_2_ < 10) or ((e_1_.a_1_ = 2 and e_1_.a_2_ < 8) or ((e_1_.a_1_ = 3 and e_1_.a_2_ < 4) or ((e_1_.a_1_ = 4 and e_1_.a_2_ < 6))

* assume the context response for entity e2 contains the following entity instances: e2_1_: {a_1_:1, a_2_:10}, e2_2_: {a_1_:2, a_2_:8}, e2_3_: {a_1_:3, a_2_:4}, e2_4_: {a_1_:4, a_2_:6}, where a1 and a2 are context attributes of entity e2.

**Table 4:** Example of execution plan

Execution Order	Entities
Execution Order 1	*schools*
Execution Order 2	*vehicles*

**Table 5:** CoaaS interface endpoints.

Address/Method	Short Description	Accepts
**/rest/cm/token (POST)**	Authentication API	Username and Password
**/rest/cm/query (POST)**	CDQL query API	• CDQL query ○ CQL ○ CDL
**/rest/cm/register/ (POST)**	Context Service registration API	CSDL Service description
**/rest/cm/event (POST)**	Context update API	Context update

**Table 6:** Context attributes of the simulated context entities for the first set of experiments.

Name	Type	Generation Strategy
**id**	String	A string value generated by concatenating “entity” and a unique number between 0 to total number of entities.
**Attribute1**	String	A string value generated by contacting “value” and a randomly generated number between 0 to 199.
**Attribute2**	Integer	Random Integer between 0 and 99.
**Location**	Geo-coordinates (i.e., latitude and longitude)	Randomly generated within a circle where the coordinates of the centre were [−37.8770, 145.0443] and the radius was 1 km.

**Table 7:** Context attributes of the simulated context entities for the second set of experiments.

Name	Type	Generation Strategy
**id**	String	A string value generated by concatenating “entity” and a unique number between 0 to total number of entities.
**joinAttr0**	String	A string value generated by concatenating “entity” and a randomly generated number between 0 to 199.
